# Surface Modification of Intumescent Flame Retardant and Its Application in Polypropylene with Excellent Fire Performance and Water Resistance

**DOI:** 10.3390/polym17030399

**Published:** 2025-02-02

**Authors:** Xuqiang Zheng, Mike Deng, Hao Jia, Xinyu Chen, Ruicheng Wang, Jun Sun, Hongfei Li, Xiaoyu Gu, Sheng Zhang

**Affiliations:** 1 State Key Laboratory of Organic-Inorganic Composites, College of Materials Science and Engineering, Beijing University of Chemical Technology, Beijing 100029, China; 2022200301@buct.edu.cn (X.Z.); c1694607530@gmail.com (M.D.); a15848142356@163.com (X.C.); 2022210198@buct.edu.cn (R.W.); hfli@mail.buct.edu.cn (H.L.); guxy@mail.buct.edu.cn (X.G.); 2 Daqing Petrochemical Research Center, Petrochemical Research Institute of PetroChina, Daqing 163714, China; jiah459@petrochina.com.cn

**Keywords:** polypropylene, intumescent flame retardant, water resistant, ammonium polyphosphate

## Abstract

Polypropylene (PP) has a wide range of applications in daily life but it is highly flammable. Intumescent flame retardants (IFRs) are used to improve the flame-retardant performance of polypropylene. However, the poor compatibility between IFRs and PP poses significant challenges. In this study, an IFR was reacted with γ-aminopropyl triethoxysilane (KH550) to introduce necessary reactive sites on the surface of the IFR. Subsequently, maleic anhydride-grafted SBS (SBS-g-MAH) was reacted with KH550 to further coat the IFR, resulting in a modified IFR named MA-IFR. The effects of MA-IFR on the flame retardancy, mechanical properties, and water resistance of PP composites were systematically investigated. The limiting oxygen index of the PP/MA-IFR composite reached up to 39.7%, with the vertical burning test (UL-94) achieving a V-0 rating. Moreover, compared to the control PP, the peak heat release rate and peak smoke release rate were reduced by 85.0% and 82.5%, respectively. In addition, the mechanical properties of the PP composites were significantly improved, with tensile strength and impact strength increasing by 29% and 18%, respectively, compared to those of the PP/IFR composite. Notably, the PP/MA-IFR composite maintained excellent flame retardancy, even after being immersed in water at 70 °C for 168 h. These results demonstrate that MA-IFR offers a promising solution for producing flame-retardant and water-resistant PP composites.

## 1. Introduction

Polypropylene (PP) is widely used across industries, such as automotive, electronics, and construction, due to its excellent mechanical properties, electrical insulating capabilities, and inherent lightweight nature [1,2,3]. However, its flammability poses a significant limitation to its broader application and development [4,5]. As a result, flame-retardant modification of PP is essential for enhancing its fire resistance [6].

Brominated flame retardants (BFRs), such as polybrominated diphenyl ethers (PBDEs), are extensively used in the market due to their high flame-retardant efficiency. However, concerns regarding their environmental impact have grown significantly [7]. Additionally, the costs associated with BFRs and antimony trioxide have been rising steadily in recent years. Common alternatives to bromine-based flame retardants include inorganic flame retardants, nanomaterials, and bio-based flame retardants. Nevertheless, these alternatives present their own challenges, such as the need for high loadings of inorganic flame retardants, which can negatively affect the mechanical properties of materials; the elevated costs of incorporating nanomaterials; and the poor stability and relatively low flame-retardant performance of bio-based flame retardants [8,9]. Intumescent flame retardants (IFRs) are highly effective, halogen-free additives that offer low smoke emission and low toxicity, making them ideal for use in PP composites [10,11]. These advantages have led to IFRs becoming one of the most widely used flame retardant systems in the PP industry [12,13]. IFRs typically consist of three key components: acid source, gas source, and carbon source [14,15]. However, the significant polarity mismatch between IFRs and PP results in poor compatibility [16,17], which may cause IFRs to migrate and precipitate over time during the use of PP composites [18]. Precipitation of IFRs can degrade the flame-retardant properties of PP composites, potentially increasing the risk of fire [19]. Therefore, it is crucial to minimize the precipitation of flame retardants in the PP matrix [20,21]. The IFRs composed of PAPP (Pyridine Phosphate) and MPP (Melamine Polyphosphate) demonstrate good compatibility with the polypropylene (PP) matrix, significantly reducing the migration of IFRs within the material [22,23]. However, this approach is relatively costly so improving the compatibility of APP-based IFRs with PP represents a cost-effective approach.

One common approach to addressing this problem is the coating method [24,25]. A reasonable coating can improve the compatibility between the IFR and PP, reduce the precipitation of the IFR in the PP matrix, and enhance the water resistance of the IFR. This modification also helps improve the mechanical properties of PP composites [26,27,28,29,30]. For example, Zhang et al. [16] developed a novel organic–inorganic hybrid flame retardant named K-HBPE@APP by coating ammonium polyphosphate (APP) with a hyperbranched polyester (HBPE) via KH-550, with a water contact angle of 75°. The excellent compatibility between K-HBPE@APP and PP significantly enhanced the mechanical and flame-retardant properties of the PP/K-HBPE@APP composite compared to those of PP/K-HBPE/APP. This highlights the effectiveness of surface coating as a strategy to strengthen the interface interaction between IFRs and polymer matrices.

In this study, the IFR was coated with a silane coupling agent (KH550), which introduced amino functional groups on the surface of the IFR. The coated IFR was then further reacted with SBS-g-MAH to complete the coating process. The SBS-g-MAH acted as the outermost protective layer, ensuring good compatibility between APP and PP. The water resistance, flame retardancy, and mechanical properties of the resulting PP composites were thoroughly evaluated. Additionally, the flame-retardant mechanism of the PP/MA-IFR composite was analyzed. This research offers a novel approach for developing PP composites with enhanced resistance to both moisture and fire.

## 2. Experimental Section

### 2.1. Materials

Polypropylene (PP, T30S) was obtained from the Sinopec Maoming Company, Maoming, China. Ammonium polyphosphate (APP, degree of polymerization ≥ 1000) was produced by Clariant Chemical Co., Ltd., Muttenz, Switzerland. Charring formation agent (CFA, consisting mainly of triazine) was provided by Guangzhou Yinyuan New Materials Co., Ltd., Suzhou, China. Additionally, γ-aminopropyl triethoxysilane (KH550) was supplied by Shanghai Macklin Biochemical Co., Ltd., Shanghai, China. Maleic anhydride-grafted SBS (SBS-g-MAH) was provided by Dongguan Shanyi Plasticization Co., Ltd., Dongguan, China. Ethyl acetate and ethanol were sourced from Beijing Chemical Works, Beijing, China.

### 2.2. Preparation of Si-IFR

A mixture of 27.0 g of intumescent flame retardant (IFR) (APP:CFA = 83:17) and 150 mL of ethanol was added to a three-neck flask equipped with a mechanical stirrer. The solution was stirred at 30 °C for 30 min. Next, 3.0 g of KH550 was added dropwise to the flask and the temperature was increased to 75 °C, maintaining stirring for 8 h. After the reaction, the mixture was washed, filtered, and dried. The resulting powder was named Si-IFR.

### 2.3. Preparation of MA-IFR

**Figure 1:**
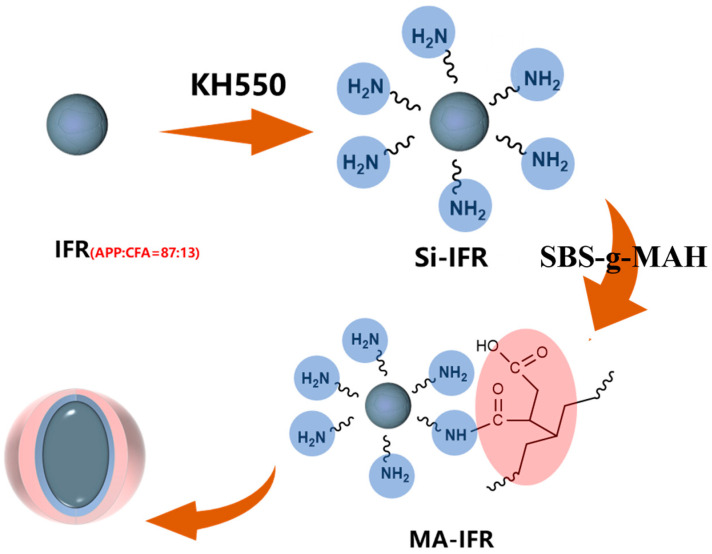
The preparation process for Si-IFR and MA-IFR.

To prepare MA-IFR, 6.0 g of SBS-g-MAH and 150 mL of ethyl acetate were added to a three-neck flask equipped with a mechanical stirrer and stirred at 50 °C until the SBS-g-MAH dissolved. Then, 27.0 g of Si-IFR was introduced into the flask and the temperature was raised to 75 °C, with stirring being continued for 5 h. Afterward, the mixture was filtered and washed three times with deionized water and anhydrous ethanol. The resulting solids were dried and named as MA-IFR. The reaction route for the preparation of Si-IFR and MA-IFR is depicted in Figure 1.

### 2.4. Preparation of MA/IFR

The preparation of MA/IFR was similar to the above procedures, with Si-IFR being replaced by IFR.

### 2.5. Preparation of PP Composites

Based on the formula provided in Table 1, the PP matrix and the flame retardant were melted and blended for 300 s at 180 °C and 50 rpm using a torque rheometer (Harbin Hapro Electric Technology Co., Ltd., Harbin, China). After blending, the PP composites were compressed at 180 °C using a flat vulcanizing machine to prepare standard samples of various sizes for subsequent testing.

### 2.6. Characterization

Fourier transform infrared (FTIR) spectra were obtained using a Nicolet IS5 infrared spectrometer (Thermo Nicolet Company, Waltham, MA, USA). FTIR spectra were recorded in the range of 400–4000 cm^−1^ using the potassium bromide tablet method, with a resolution of 1 cm^−1^ and 32 scans.

Thermogravimetric analyses (TGA)were performed using a TA-Q50 thermogravimetric analyzer (TA Instruments, Newcastle, WA, USA). Samples (3–5 mg) were heated from 30 °C to 800 °C at a rate of 10 °C/min in a nitrogen atmosphere.

X-ray photoelectron spectroscopy (XPS) was performed using the ESCALAB 250 system (Thermo Fisher Scientific, Waltham, MA, USA), with an Al Kα X-ray source, a voltage of 284.8 eV, and a step size of 0.5 eV.

Scanning electron microscopy (SEM) images and energy-dispersive X-ray spectroscopy (EDS) were determined using a HITACHI S-4700 instrument (JEOL, Tokyo, Japan), at an acceleration voltage of 20 kV.

Contact angle measurements were conducted using a Data Physics OCA20 system (DataPhysics Instruments GmbH, Stuttgart, Germany) with disk-shaped samples and 3 μL droplets of deionized water.

The limiting oxygen index (LOI) was determined using a JF3 oxygen index apparatus (ISO 4589 [31], Nanjing Jiangning Analytical Instrument Co., Ltd., Nanjing, China), with sample dimensions of 100 × 6.5 × 3.2 mm^3^. At least five parallel samples were tested for each specimen to obtain an average value.

The UL-94 vertical combustion test was conducted with a Jiangning CZF-3 apparatus (ISO 9773 [32], Nanjing Jiangning Analytical Instrument Co., Ltd., Nanjing, China). The sample size was 100 × 13 × 3.2 mm^3^ and the average value was taken from five parallel samples.

Cone calorimeter testing (CCT) was performed using a cone calorimeter (CONE) (ISO 5660 [33], Fire Testing Technology, East Grinstead, UK). The sample size was 100 × 100 × 3 mm^3^ and the irradiation intensity was set at 35 kW/m^2^. The average value was calculated from two parallel samples.

The mechanical properties were measured using a CMT4104 Tensile Testing Machine (ASTM D256 [34], SANS Co., Ltd., Shenzhen, China) and an XJJ-5 Impact Tester (ISO 527 [35], Chengde Jinjian Testing Instrument Co., Ltd., Chengde, China). The tensile test samples had dimensions of 100 × 13 × 3 mm^3^ while the impact test specimens measured 64 × 13 × 3 mm^3^. All tests were conducted at room temperature. The average values were calculated from two parallel samples for each test.

The samples were analyzed using a LabRam HR Evolution High-Resolution Raman Spectrometer (HORIBA Scientific, Kyoto, Japan) within the wavelength range of 1800–500 cm^−1^, with an excitation wavelength of 514 nm.

The water resistance of the PP composite material was assessed by evaluating both its fire performance and mechanical properties before and after immersion. The specimens were soaked in water at 70 °C for 7 days and then placed in a vacuum oven at 70 °C until their weight remained stable.

## 3. Results and Discussion

### 3.1. Characterizations of IFR, Si-IFR, MA-IFR, and MA/IFR

**Figure 2:**
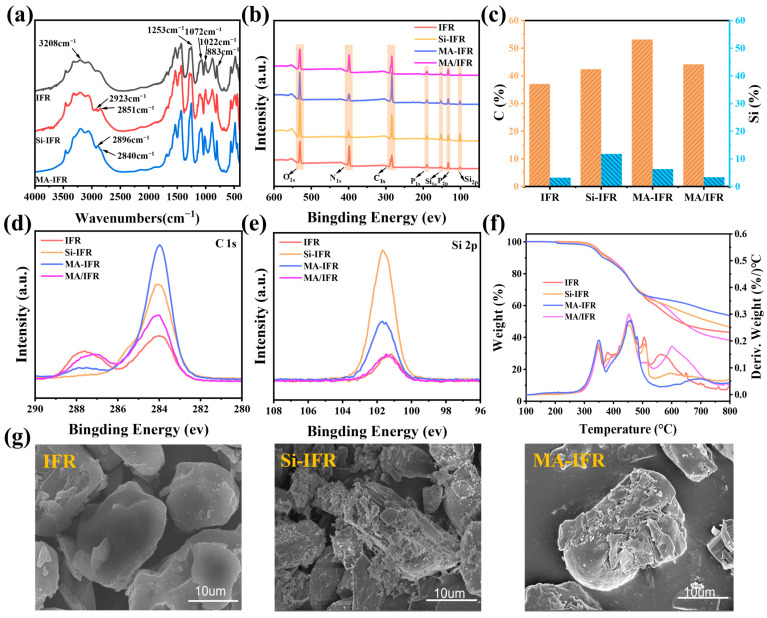
FTIR (**a**); XPS spectra (**b**); bar chart of variation in C and Si element contents (**c**); C 1s spectra (**d**) and Si 2p spectra (**e**) of XPS spectra; TG and DTG curves under N_2_ atmosphere (**f**); SEM images (**g**).

The FTIR spectra of the IFR, Si-IFR, and MA-IFR are shown in Figure 2a. The spectra of the IFR were consistent with those reported in the literature, with typical absorption peaks observed at 3208 cm^−1^ (N–H), 1253 cm^−1^ (P=O), 1072 cm^−1^ (P–O), 1022 cm^−1^ (PO_2_), and 883 cm^−1^ (PO_3_) [5]. After modification with KH550, both Si-IFR and MA-IFR displayed new absorption bands at 2923 and 2851 cm^−1^, corresponding to the stretching vibrations of C–H in the methyl and methylene groups, respectively [36,37]. Additionally, compared to Si-IFR, the absorption peaks for the methyl and methylene groups in MA-IFR shifted to 2896 and 2840 cm^−1^, with a slight increase in absorption peak intensity. These changes suggested that KH550 and SBS-g-MAH successfully coated the IFR.

To further verify the coating of the IFR surface, XPS analysis was conducted and the results are presented in Figure 2b–e. Compared to the IFR, the Si content on the surface of Si-IFR increased significantly while the C content decreased, indicating successful coating of the IFR particles with KH550. Furthermore, compared to Si-IFR, the Si content on the surface of MA-IFR decreased and the C content increased, suggesting that the SBS-g-MAH shell was successfully applied to the Si-IFR surface. Additionally, MA-IFR showed higher Si and C content than MA/IFR, further confirming that KH550 provided reactive sites that facilitated the attachment of SBS-g-MAH to the surface of the IFR particles.

The surface morphology of the IFR, Si-IFR, and MA-IFR is shown in Figure 2g. The surface of the unmodified IFR appeared relatively smooth while the Si-IFR exhibited a rougher surface, likely due to the KH550 adhering to the particle surface [4]. After the further coating of SBS-g-MAH, the attachments on the surface of MA-IFR became larger than those on Si-IFR, providing evidence of the successful coating of SBS-g-MAH.

Thermal stability before and after modification was investigated using a TGA. The corresponding results are shown in Figure 2f and the detailed data are listed in Table 2. IFR, Si-IFR, MA-IFR, and MA/IFR all demonstrated good thermal stability, with their initial decomposition temperatures (T_5%_) exceeding 180 °C, which was well above the processing temperature of the PP matrix [16]. The residual char content of the IFR at 800 °C was 43.2 wt.%, indicating its excellent thermal stability and charring ability at high temperatures. Compared to unmodified IFR, MA/IFR exhibited an increased peak thermogravimetric rate after 530 °C and a reduced char residue of 38.3 wt.% at 800 °C. This decrease in char residue could be attributed to the breakdown of SBS-g-MAH on the IFR surface at high temperatures. In contrast, the char residue rates of Si-IFR and MA-IFR at 800 °C were 46.6 wt.% and 54.3 wt.%, respectively, and both exhibited a decrease in thermogravimetric rates after 530 °C. These results suggested that the introduction of Si improved the thermal stability of the IFR, while SBS-g-MAH, in conjunction with the silane, facilitated the formation of more stable char layers on the IFR at elevated temperatures.

**Figure 3:**
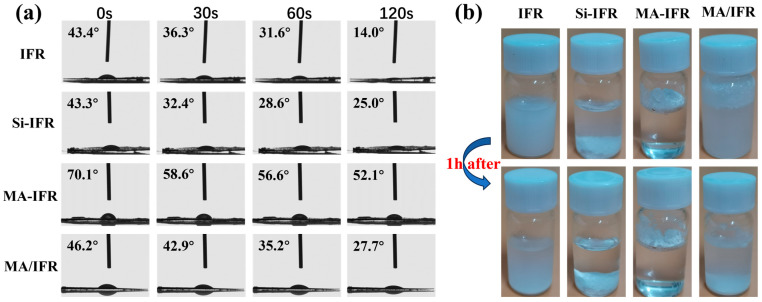
The water contact angles (**a**); dispersion in water (**b**).

The hydrophilicity of the IFR before and after modification was evaluated using the water contact angle (WCA) test. As shown in Figure 3a, the WCA value of unmodified IFR was 43.4°, which decreased to 14° after 120 s, indicating that the IFR was hydrophilic and readily absorbed water. Similarly, the WCA values for Si-IFR and MA/IFR were 43.3° and 46.2°, respectively, and both values decreased to 25.0° and 27.7° after 120 s. In contrast, the WCA value of MA-IFR increased to 70.1° and remained stable at 52.1° after 120 s. These results suggested that SBS-g-MAH successfully coated the IFR and reduced its surface polarity.

To further illustrate the changes in water resistance before and after modification, 1 g of the IFR particles (both before and after modification) was placed in a glass bottle and 10 mL of deionized water was added. After shaking for 15 min, the mixture was allowed to stand for 1 h to observe the dispersion of the particles in water. As shown in Figure 3b, uncoated IFR particles were fully immersed in deionized water and the liquid remained cloudy after 1 h, demonstrating the strong hydrophilicity of the unmodified IFR. After being coated with KH550, Si-IFR showed different behavior. After standing for 1 h, Si-IFR settled at the bottom of the glass bottle and the water became clear and transparent. This suggested that the hydrophobicity of Si-IFR was improved. After further coating with SBS-g-MAH, MA-IFR remained suspended on the surface of the deionized water and was insoluble, indicating that it was more hydrophobic. In contrast, the test results for MA/IFR were similar to those for unmodified IFR, implying that no effective chemical bonding occurred between the IFR and SBS-g-MAH. The results from these experiments confirmed that MA-IFR exhibited lower hydrophilicity than unmodified IFR. This could be attributed to the SBS-g-MAH coating, which significantly reduced the surface polarity of the IFR. The reduction in surface polarity enhanced the compatibility of the flame retardant with the PP matrix [38].

### 3.2. Thermal Stability of PP and PP Composites

**Figure 4:**
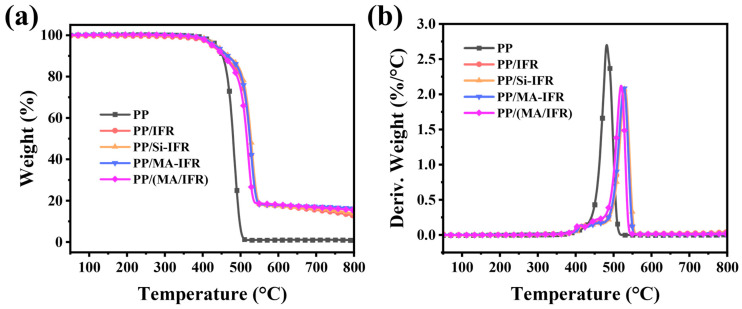
The (**a**) TG curves and (**b**) DTG curves under the N_2_ atmosphere.

To investigate the thermal stability of PP and its composites, TGA tests were conducted. The results are shown in Figure 4 and Table 3. T_max1_ and T_max2_ corresponded to the temperatures at which the maximum thermogravimetric rate occurred during different stages of decomposition. It could be observed that pure PP exhibited a single thermal decomposition peak, with T_5%_ and T_max1_ occurring at 437.0 °C and 482.0 °C, respectively. At 800 °C, the char residue of PP was 0 wt.%. For the PP/IFR composite, the T_5%_ value shifted to 424.1 °C, which could be attributed to the earlier decomposition of the IFR. Additionally, two distinct decomposition peaks appeared, with T_max1_ and T_max2_ observed at 412.4 °C and 526.7 °C, respectively, and the char residue increased to 12.8 wt.% [26]. For the PP/Si-IFR composite sample, the thermal stability was improved, as evidenced by the slightly increased T_5%_, T_max1_, and T_max2_ values of 435.5 °C, 421.3 °C, and 530.1 °C, respectively. The char residue also increased to 13.8 wt.%, indicating that KH550 enhanced the thermal stability of the composites. In contrast, the T_5%_, T_max1_, and T_max2_ values of the PP/MA-IFR and PP/(MA/IFR) composites shifted to lower temperatures, with PP/(MA/IFR) showing the most significant decrease. This was likely due to the early decomposition of SBS-g-MAH at high temperatures. At 800 °C, the char residue of the PP/MA-IFR and PP/(MA/IFR) samples increased to 16.3 wt.% and 15.5 wt.%, respectively. The theoretical residual char was obtained based on the hypothesis that there is no reaction between the PP matrix and flame retardant additives. For all the PP composites, the experimental residual char was higher than the theoretical value, indicating the existence of synergism [39].

### 3.3. Water Resistance and Flame Retardancy of PP and PP Composites

Table 4 presents the changes in the UL-94 rating and LOI values of PP composites before and after the water immersion test. Prior to the water immersion test, all samples exhibited good flame retardancy, achieving a V-0 rating in the UL-94 test. Notably, the PP/MA-IFR composite showed the highest LOI value (39.7%), indicating that SBS-g-MAH effectively enhanced the flame-retardant properties of the PP composites. After the water immersion test at 70 °C for 168 h, the LOI values of all PP composites decreased and the UL-94 rating of the PP/IFR sample dropped from V-0 to V-2. However, the samples containing MA-IFR still maintained a V-0 rating. Compared to the PP/IFR composite, the PP/Si-IFR composite exhibited lower ΔLOI values, suggesting that the introduction of KH550 improved the water resistance of the sample [21]. The PP/MA-IFR composite showed the smallest ΔLOI value, indicating that the incorporation of SBS-g-MAH further enhanced the water resistance of the composite. This improvement could be attributed to the long-chain macromolecule structure of SBS-g-MAH, which not only improved the water resistance of the IFR but also enhanced the compatibility between the IFR and PP. As a result, the migration of the IFR within the PP matrix was inhibited, leading to better water resistance in the PP composite. Conversely, the PP/MA/IFR composite did not exhibit any significant improvement in water resistance.

### 3.4. Flame Performance of PP and PP Composites

**Figure 5:**
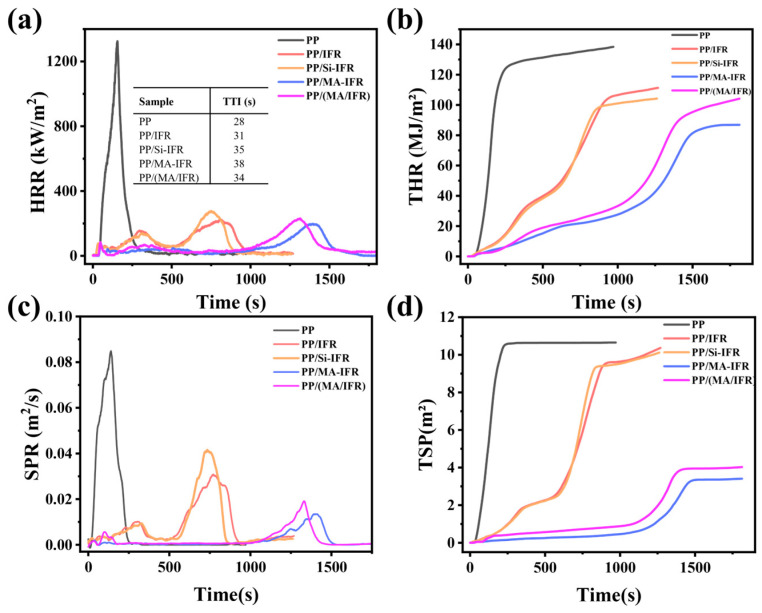
HRR (**a**), THR (**b**), SPR (**c**), and TSR (**d**) curves of the cone calorimetry testing of PP and its composites.

Cone calorimeter tests (CCTs) were conducted to further assess the flame-retardant properties of the PP composites and the results were presented in Figure 5 and Table 5. The initial ignition time (TTI) was a key parameter for evaluating the fire resistance of polymers [40]. The TTI of the pure PP sample was 28 s, indicating its high susceptibility to ignition. Upon the introduction of the IFR, the TTI of the PP/IFR composite increased to 31 s. The TTI values of the PP/Si-IFR, PP/MA-IFR, and PP/(MA/IFR) composites all increased to varying extents, with PP/MA-IFR exhibiting the greatest improvement. In addition, the peak heat release rate (pHRR) and total heat release (THR) were critical parameters for assessing the fire hazard of polymer composites [41]. The pHRR and THR of the pure PP sample were 1325 kW/m^2^ and 138 MJ/m^2^, respectively. The pHRR and THR of the PP/IFR composite decreased to 266 kW/m^2^ and 110 MJ/m^2^, respectively. In comparison to the PP/IFR composite, the pHRR and THR of the PP/MA-IFR composite were further reduced to 198 kW/m^2^ and 87 MJ/m^2^. This suggested that the combination of KH550 and SBS-g-MAH had a synergistic effect, significantly reducing the THR of the PP composites and enhancing their flame-retardant performance. In terms of time to peak heat release (T_pHRR_), the values for the PP/IFR, PP/Si-IFR, PP/MA-IFR, and PP/(MA/IFR) composites were extended to 755 s, 691 s, 1392 s, and 1315 s, respectively. As shown in Figure 5c,d, the total smoke production (TSP) and the peak smoke production rate (pSPR) of PP/Si-IFR showed no significant changes compared to those of the PP/IFR composite. However, the TSP and pSPR of the PP/(MA/IFR) composite were significantly reduced to 4.7 m^2^ and 0.021 m^2^/s, respectively. This indicated that the SBS-g-MAH shell played a crucial role in reducing flue gas emissions. This could be attributed to SBS-g-MAH acting as a high-quality carbon source, which, in conjunction with the IFR, promoted the formation of a char layer in the PP matrix at high temperatures. This char layer acted as a barrier, preventing smoke from escaping. Compared to the PP/(MA/IFR) composite, the TSP and pSPR of the PP/MA-IFR composite were further reduced by 28% and 19%, respectively. This further improvement could be attributed to the synergistic effect of KH550 with SBS-g-MAH, which enhanced the density of the char layer, thereby providing better protection against flame and smoke release.

### 3.5. Char Residue Analysis

**Figure 6:**
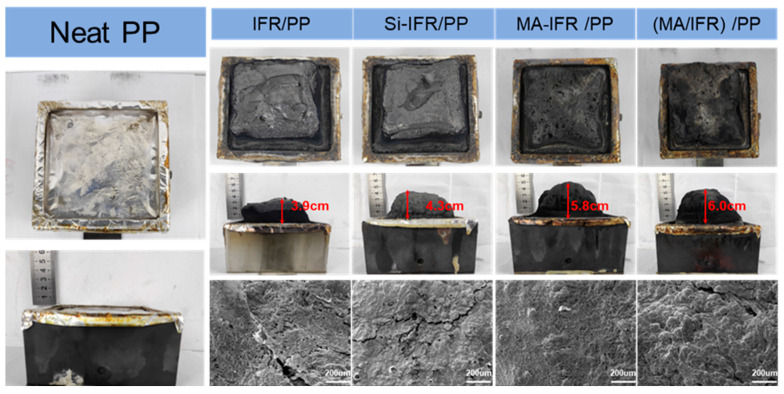
Digital photos and corresponding SEM images of residual char after cone calorimeter testing.

Intumescent flame retardant systems typically underwent a rapid expansion process, forming a protective char layer. Therefore, investigating the char residue was crucial for understanding changes in flame retardancy [42]. Figure 6 presents digital photos and SEM images of the char residue of PP and its composites after cone calorimeter testing. It was evident that PP produced no residual char. Upon adding 27 wt.% IFR, the char residue of the PP/IFR composite increased to 14.2 wt.% and the height of the expanded char layer reached 3.9 cm, demonstrating the good char-forming ability of the IFR. The char residue of the PP/Si-IFR composite increased to 14.7 wt.% and the char layer height grew to 4.3 cm, indicating that KH550 enhanced the charring process. The char residue of the PP/(MA/IFR) composite rose to 19.4 wt.%, with the char layer height increasing to 6.0 cm. Notably, the char residue of the PP/MA-IFR composite was much higher while the char layer height remained almost unchanged, indicating the formation of a denser char layer.

Furthermore, SEM analysis of the surface morphology of the char residues revealed that the char layer of the PP/MA-IFR composite was more intact and cohesive than those of the other samples. These results suggested that the PP/MA-IFR composite generated a more complete and dense char residue during combustion, which effectively isolated the release of combustible gases and smoke. Additionally, the dense char layer acted as a protective barrier, preventing further combustion of the PP matrix.

**Figure 7:**
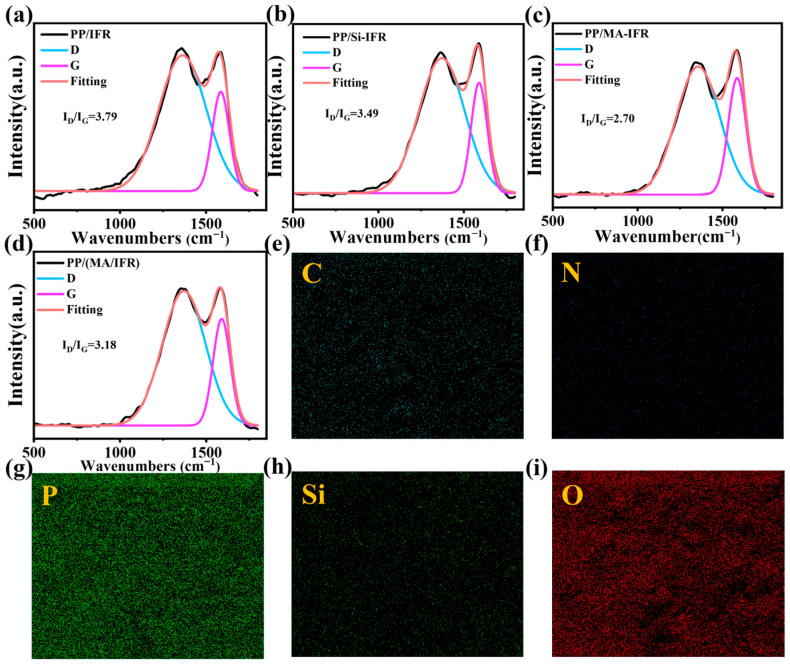
Raman spectroscopy of PP and its composites (**a**–**d**); EDS images of the char residue of PP/MA-IFR (**e**–**i**).

The residual char of PP composites was analyzed using Energy Dispersive X-ray Spectroscopy (EDS) and Raman spectroscopy, with the results presented in Figure 7. According to the EDS analysis, the presence of silicon (Si) on the residual char surface of the PP/MA-IFR composite indicated that KH550 participated in the charring process.

Raman spectroscopy was employed to assess the degree of graphitization of the residual char in the PP composites. The D-band and G-band peaks appeared at 1360 cm^−1^ and 1600 cm^−1^, respectively. By applying Gaussian fitting to calculate the peak areas, the I_D_ and I_G_ ratios were determined. The I_D_/I_G_ ratio was inversely related to the degree of graphitization and lower values indicated higher graphitization [43]. The I_D_/I_G_ ratios for the residual char in the PP composites were as follows: PP/IFR (3.79) > PP/Si-IFR (3.49) > PP/(MA/IFR) (3.18) > PP/MA-IFR (2.70), suggesting that the PP/MA-IFR composite exhibited the highest degree of graphitization. These findings indicated that KH550 and SBS-g-MAH worked synergistically to enhance the graphitization of the residual char, promoting the formation of a more stable and well-structured char layer.

### 3.6. Flame Retardant Mechanism

**Figure 8:**
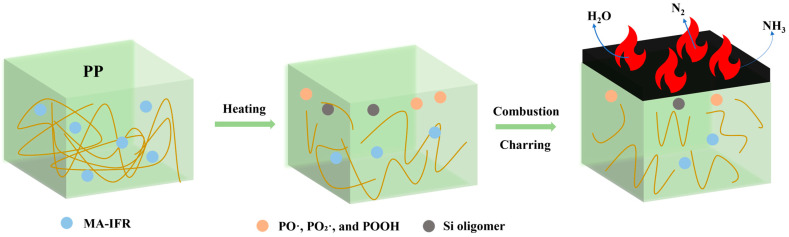
Possible flame-retardant mechanism of the PP/MA-IFR composite.

Based on the test results above, a char formation mechanism for the PP/MA-IFR composites was proposed, as shown in Figure 8. According to previous studies [5], the IFR decomposes during the combustion of PP composites, producing non-combustible gases, such as NH_3_, H_2_O, and N_2_, as well as reactive species, like PO, PO_2_, and POOH. Some of them contributed to the formation of an intumescent char layer through the combined action of carbon and gas sources. For the PP/MA-IFR composite, EDS analysis of the residual char indicated that silicon (Si) played a role in the char layer formation. This might be attributed to the high-temperature reaction between KH550 and POOH generated during IFR decomposition, resulting in the formation of Si-OH oligomers and Si-O-C structures. These interactions enhanced the stability of the char layer. Additionally, during the initial thermal decomposition of the IFR, SBS-g-MAH in the MA-IFR shell acted as a superior carbon source, promoting the early formation of the char layer and enhancing both its height and density. The synergistic effect of SBS-g-MAH and KH550 thus resulted in a higher degree of graphitization in the residual char of the PP/MA-IFR composite, which effectively prevented the exchange of external oxygen and internal combustible gases during combustion. This led to less smoke and heat release, thereby enhancing the flame-retardant properties of the composite.

### 3.7. Mechanical Properties of PP and PP Composites

**Figure 9:**
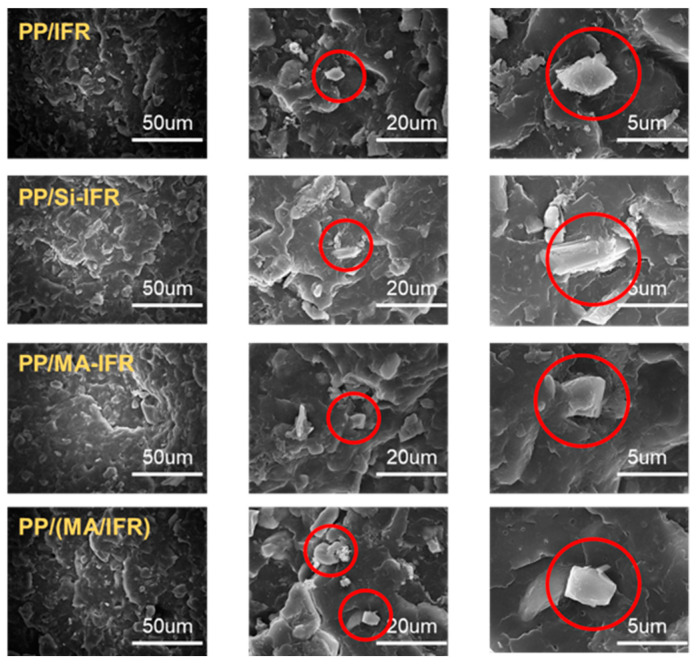
SEM images of cross-sections of different PP composites.

PP materials are known for their excellent mechanical properties but the incorporation of IFRs, which typically exhibit weak interfacial interactions, often leads to a deterioration in the mechanical performance of the PP matrix [2]. To assess this, mechanical tests were conducted on the PP composites; the results are shown in Figure 9 and Table 6.

When unmodified IFR was introduced into the PP matrix, the resulting PP/IFR composite exhibited relatively poor mechanical properties: the tensile strength was only 21.4 MPa, the elongation at break was 6.3%, and the impact strength was 1.92 kJ/m^2^. However, the mechanical strength of the PP/Si-IFR composite showed improvement. In addition, it was noticeable that the tensile strength, elongation at break, and impact strength of the PP/MA-IFR composite were significantly enhanced, reaching values of 27.7 MPa, 15.4%, and 2.27 kJ/m^2^, respectively. These improvements could be attributed to the introduction of SBS-g-MAH, which enhanced the interfacial compatibility between the IFR and the PP matrix, thereby improving the dispersion of the IFR within the matrix. In contrast, the mechanical properties of the PP/(MA/IFR) composite were found to be inferior. This was likely due to the lack of stable interactions between SBS-g-MAH and the IFR, leading to phase separation during processing. As a result, the flame retardant was poorly dispersed throughout the matrix, which negatively impacted the overall mechanical performance of the composite.

To further investigate the distribution of the IFR within the PP matrix before and after modification, SEM images of the cross-sectional structure of the PP composites were analyzed (Figure 9). In the PP/IFR composite, numerous voids were visible, and the flame retardant (highlighted in the red circle) appeared poorly distributed and incompatible with the PP matrix. This was likely due to the significant polarity difference between the IFR and PP, leading to weak intermolecular forces at the interface. For the PP/Si-IFR composite, the pores were reduced compared to those of the PP/IFR composite. This suggested that the silane coating helps to improve the dispersion of the flame retardant within the PP matrix. Furthermore, the voids and gaps on the cross-sectional surface of PP/MA-IFR were further reduced, with almost no visible interface between the filler and the matrix. This indicated that the flame-retardant particles were effectively embedded in the PP matrix. Conversely, the fracture surface of the PP/(MA/IFR) composite was similar to that of the PP/IFR composite, showing poor dispersion and compatibility. Comparing the SEM images before and after modification revealed that the introduction of the long-chain macromolecule SBS-g-MAH significantly improved the compatibility and dispersion of the IFR particles within the PP matrix.

## 4. Conclusions

In this study, we successfully synthesized MA-IFR through the coating of KH550 and the chemical reaction of SBS -g-MAH. Due to the existence of KH550, the water contact angle of MA-IFR increased to 52.1° after 120 s, indicating the significant enhancement of the water resistance. Furthermore, when MA-IFR was added to the PP matrix, the PP/MA-IFR exhibited excellent flame retardancy, with the LOI value of 39.7%, V-0 rating, and reductions of 85% and 82.5% in pHRR and pSPR, respectively, compared to those of the pure PP sample. In addition, it was noticeable that the PP/MA-IFR composite displayed a lower ΔLOI than that of the other composites and maintained a V-0 rating after the water immersion test. Moreover, the PP/MA-IFR composite showed outstanding mechanical properties in terms of tensile and impact strengths. In summary, this study developed a PP composite material with both flame-retardant and water-resistant properties and it is expected to have broad application prospects in household appliances, such as washing machine casings and air conditioner outdoor units.

## Figures and Tables

**Table 1:** Formulations of PP and its composites.

Samples	PP (wt.%)	IFR (wt.%)	Si-IFR (wt.%)	MA-IFR (wt.%)	MA/IFR (wt.%)
PP	100	0	0	0	0
PP/IFR	73	27	0	0	0
PP/Si-IFR	73	0	27	0	0
PP/MA-IFR	73	0	0	27	0
PP/(MA/IFR)	73	0	0	0	27

**Table 2:** Thermal degradation data of the IFR, Si-IFR, MA-IFR, and MA/IFR.

Samples	T_5%_ (°C)	T_max_ (°C)	Residual Char (wt.%)
IFR	347	456	43.2
Si-IFR	344	453	46.6
MA-IFR	337	456	54.3
MA/IFR	338	453	38.3

**Table 3:** Key data in the TGA and DTG curves of PP and its composites.

Samples	T_5%_ (°C)	T_Max1_ (°C)	T _Max2_ (°C)	Residual Char (wt.%)	
				Calculated	Experimental
PP	437.0	482.0	-	-	0.0
PP/IFR	424.1	412.4	526.7	11.7	12.8
PP/Si-IFR	435.5	421.3	530.1	12.6	13.8
PP/MA-IFR	433.7	413.9	525.5	14.7	16.3
PP/(MA/IFR)	426.8	412.6	519.7	10.3	15.5

**Table 4:** Changes in the flame-retardant performance of PP and its composites before and after the water immersion test.

Sample	UL-94_Before boiling_		UL-94_After boiling_		LOI_Before boiling_ (%)	LOI_After boiling_ (%)	ΔLOI (%)
	(t1 + t2)/s	Rating	(t1 + t2)/s	Rating			
PP/IFR	0.2	V-0	4.3	V-2	37.5 ± 0.3	31.5 ± 0.3	6.0
PP/Si-IFR	0.1	V-0	2.2	V-0	37.5 ± 0.3	33.2 ± 0.3	4.3
PP/MA-IFR	0.2	V-0	1.8	V-0	39.7 ± 0.3	35.5 ± 0.3	4.2
PP/(MA/IFR)	0.2	V-0	3.3	V-2	38.4 ± 0.3	32.5 ± 0.3	5.9

**Table 5:** Key data on PP and its composites in cone calorimetry testing.

Sample	TTI (s)	pHRR (kW/m^2^)	T_pHRR_ (s)	THR (MJ/m^2^)	TSP (m^2^)	pSPR (m^2^/s)	Residual Mass (wt.%)
PP	28	1325 ± 41	129 ± 4	138 ± 5	10.6 ± 0.3	0.097 ± 0.004	0
PP/IFR	31	266 ± 8	755 ± 22	110 ± 4	7.1 ± 0.2	0.034 ± 0.001	14.2 ± 0.4
PP/Si-IFR	35	227 ± 8	691 ± 21	104 ± 4	10.1 ± 0.3	0.049 ± 0.002	14.7 ± 0.4
PP/MA-IFR	38	198 ± 6	1392 ± 42	87 ± 3	3.4 ± 0.1	0.017 ± 0.001	25.9 ± 1
PP/(MA/IFR)	34	231 ± 7	1315 ± 40	108 ± 4	4.7 ± 0.1	0.021 ± 0.001	19.4 ± 0.8

**Table 6:** Key data on the tensile strength testing and impact strength testing of PP composites.

Sample	Tensile Strength (MPa)	Elongation at Break (%)	Impact Strength (KJ/m^2^)
PP/IFR	21.4 ± 1.4	6.3 ± 1.0	1.92 ± 0.06
PP/Si-IFR	23.1 ± 1.5	11.6 ± 0.7	2.05 ± 0.04
PP/MA-IFR	27.7 ± 1.4	15.4 ± 0.4	2.27 ± 0.02
PP/(MA/IFR)	18.3 ± 3.1	6.3 ± 1.0	2.15 ± 0.12

## Data Availability

The original contributions presented in this study are included in the article. Further inquiries can be directed to the corresponding authors.
